# Abiraterone-Docetaxel scheduling for metastatic castration-resistant prostate cancer based on evolutionary dynamics

**DOI:** 10.1371/journal.pone.0282646

**Published:** 2023-03-09

**Authors:** Atefeh Deris, Mahdi Sohrabi-Haghighat

**Affiliations:** Faculty of Science, Arak University, Arak, Iran; Adana Alparslan Turkes Science and Technology University: Adana Alparslan Turkes Bilim ve Teknoloji Universitesi, TURKEY

## Abstract

Patients with metastatic castration-resistant prostate cancer (mCRPC) are divided into three groups based on their response to Abiraterone treatment: best responder, responder, and non-responder. In the latter two groups, successful outcomes may not be achieved due to the development of drug-resistant cells in the tumor environment during treatment. To overcome this challenge, a secondary drug can be used to control the population of drug-resistant cells, potentially leading to a longer period of disease inhibition. This paper proposes using a combination of Docetaxel and Abiraterone in some polytherapy methods to control both the overall cancer cell population and the drug-resistant subpopulation. To investigate the competition and evolution of mCRPC cancer phenotypes, as in previous studies, the Evolutionary Game Theory (EGT) has been used as a mathematical modeling of evolutionary biology concepts.

## Introduction

Prostate cancer is a common cancer among men and the second-leading cause of cancer death in men [1]. Early diagnosis is possible through Prostate-Specific Antigen (PSA) testing, improving men’s life expectancy [2]. High levels of androgens are associated with the development and progression of prostate cancer in men. CYP17A, AR, SRD5A2, HSD3B1, and HSD3B2 genes play a special role in androgen metabolism and cell proliferation in the prostate [3]. Androgen deprivation therapy (ADT) is used to slow the growth of prostate cancer by reducing the androgen concentration in blood or preventing androgens from affecting the prostate cancer cells [4]. The CYP17A enzyme is involved in androgen synthesis and the pathogenesis of prostate cancer. Inhibitors of CYP17A, such as abiraterone, stop the production of androgens by prostate cancer cells by inhibiting the CYP17A enzyme in the prostate [5, 6].

Metastatic castration-resistant prostate cancer can still grow and spread despite reduced testosterone production [7, 8]. There are three phenotypes of mCRPC: *T*^+^, *T*^*P*^, and *T*^−^. *T*^+^ cells require testosterone to survive and multiply, while *T*^*P*^ cells produce the necessary testosterone through rearrangement of CYP17A. *T*^−^ cells, which are resistant, can multiply without relying on testosterone [9, 10].

One common treatment for mCRPC is the use of maximum tolerated dose (MTD) treatment, in which medications are used at their highest tolerable dose as long as there are no side effects. However, the MTD treatment approach can lead to the elimination of sensitive phenotypes and the competitive release of the resistant population, which can lead to the development of resistance and the failure of treatment [11, 12].

An alternative approach is adaptive therapy, which is a personalized treatment method aimed at long-term control of cancer, and has shown promising results [13–18]. Zhang et al. [19] applied adaptive therapy to ADT by using Abiraterone when the PSA level was more than 50% of the pretreatment value, and stopping treatment if the PSA level dropped below 50% of the pretreatment value. Adherence to this simple model resulted in a longer duration of cancer inhibition compared to the traditional Maximum Tolerated Dose (MTD) approach. In addition, the Adaptive Therapy (AT) used a significantly lower dose of Abiraterone, approximately half the amount used in the MTD method.

This trial prompted further studies and investigations to analyze and interpret the significant improvement in disease control and patient survival, with a focus on the application of Evolutionary Game Theory [14, 17, 18, 20–25]. Evolutionary Game Theory is a subfield of game theory that combines game theory and evolutionary biology and has been applied in cancer research.

In the heterogeneous environment of cancer, different cancer cell phenotypes compete for common resources and survival. EGT models the competition between cancer cells, providing insight into the interactions between cells and the factors that influence the competition. Anti-cancer drugs target dominant phenotypes, but this can lead to the spread of drug-resistant phenotypes. EGT considers both the use of drugs and the balance between drug-sensitive and drug-resistant cells to address this issue and improve our understanding of cancer evolution and the development of new cancer therapies. By improving the combination of treatments and understanding the conditions under which resistance is likely to evolve, EGT has the potential to improve cancer treatment outcomes and inhibit cancer over the long term [15, 25–27].

mCRPC patients undergoing Abiraterone therapy can be categorized into three groups: best responder, responder, and non-responder, based on both theoretical and practical considerations. The clinical trial [19] found that Abiraterone treatment was only effective in preventing the development of drug-resistant cells in the best responder group among mCRPC patients. In later studies [14, 23], the administration schedule of Abiraterone was altered in an effort to prevent the dominance of drug-resistant cells, but these attempts were unsuccessful in both the responder and non-responder groups. Another study [24] suggested that adding Docetaxel to the treatment may increase patient survival time, however, the analysis was limited to the best responder group only. Docetaxel is a chemotherapy drug used to treat various cancers and works by promoting the assembly and stabilizing microtubules while preventing disassembly and depolymerization in the absence of Guanosine-5’-triphosphate [28].

In this paper, we examine the mechanism of drug effects on mCRPC and develop methods for using a second drug to treat responder and non-responders to initial Abiraterone therapy. The addition of the second drug helps control tumor volume and maintain a balance between different phenotypes, preventing the dominance of drug-resistant cells.

## Method and model

One of the common ways to treat mCRPC is with ADT using Abiraterone, which reduces the amount of testosterone needed by cancer cells. The use of Abiraterone effectively lowers the number of *T*^+^ and *T*^*P*^ cells, but does not impact *T*^−^ cell growth [10]. As a result, *T*^−^ cells may proliferate and comprise a larger portion of the cancer cell population, potentially leading to treatment failure. Therefore, during Abiraterone treatment, it is advisable to focus on phenotype growth rather than PSA-based scheduling, which is based on the total number of cancer cells, to achieve successful results.

The interaction and competition among phenotypes *T*^+^, *T*^*P*^, and *T*^−^ can be modeled using a 3 × 3 payoff matrix. According to You et al. [9], there are 22 modes for the payoff matrix entries of the phenotypes *T*^+^, *T*^*P*^, and *T*^−^. These 22 modes can be divided into three groups based on the frequency of resistant *T*^−^ cells in evolutionarily stable strategies (ESS): best responder (zero frequency of *T*^−^ phenotype), responder (frequency between 0 and 15% of *T*^−^ phenotype), and non-responder (frequency over 15% of *T*^−^ phenotype). ESS is a strategy in EGT that cannot be replaced by any other strategy if widely adopted in a population. The study of ESS helps explain the evolution of behaviors and strategies in populations and provides insight into the stability of cooperative and competitive behaviors [29].

References [14, 19, 23] indicate that appropriate scheduling with Abiraterone leads to an effective treatment in the “best responder” group, while in the “responder” and “non-responder” groups, the Abiraterone treatment results the competitive release and growth of *T*^−^ cells. To control resistant cell growth, we introduced Docetaxel into the treatment. The combination of Abiraterone and Docetaxel may result in the emergence of a new phenotype resistant to both drugs, represented as *T*^−−^.

West et al. [24] improved outcomes in the “best responder” group through a combination therapy of Abiraterone and Docetaxel that was based on PSA biomarker levels. Here, we focus on the responder and non-responder groups. By using evolutionary game theory and considering the replicator equation of each cancer phenotype, especially drug-resistant phenotypes [30, 31], we present combination therapies of Abiraterone and Docetaxel that reduce the total number of cancer cells and resistant phenotypes to an acceptable range at the end of treatment.

Table 1 shows the payoff matrix A, which consists of 4 phenotypes. The competition coefficients *a*_*ij*_ estimate the impact of cell type *j* on the growth rate of cell type *i*. They are qualitative estimates, and the relative values determine the evolution of phenotypes, not the absolute values. The intracellular coefficients are set to 1, while the relative values for other coefficients are obtained from prostate oncologists and prior research. The relationships between the matrix entries corresponding to phenotypes *T*^+^, *T*^*P*^, and *T*^−^ are established as follows: *a*_31_ > *a*_21_, *a*_32_ > *a*_12_, *a*_13_ > *a*_23_, *a*_13_ > *a*_12_, *a*_23_ > *a*_21_, and *a*_32_ > *a*_31_ [19, 23, 24]. The values in the fourth row and column of matrix A are lower than those in the third row and column, respectively, due to the cost of resistance, except for *a*_44_ which is equal to 1 because *T*^−−^ cells in this position compete with cells of the same type.

**Table 1:** The payoff matrix A consist of 4 phenotypes.

	*T* ^+^	*T* ^*P*^	*T* ^−^	*T* ^−−^
*T* ^+^	1	*a*_12_	*a*_13_	*a*_14_
*T* ^*P*^	*a*_21_	1	*a*_23_	*a*_24_
*T* ^−^	*a*_31_	*a*_32_	1	*a*_34_
*T* ^−−^	*a*_41_	*a*_42_	*a*_43_	1

We selected a sample competition matrix of each responder and non-responder groups. The selected values in this paper are chosen based on comparable studies on the same disease for comparability and understanding of the results. Therefore, samples, initial values, parameters and coefficients are selected based on previous studies such as [19] which used Abiraterone therapy with 50% PSA criterion on triple groups, [14, 23] which used Abiraterone therapy without considering the 50% PSA criterion on triple groups, and [24] which used a combination of Abiraterone and Docetaxel only on the best responder group. Table 2 provides the entries of the competition matrices and Table 3 gives the initial values of each subpopulation. As indicated in Table 3, the initial subpopulation of phenotype *T*^−−^ is estimated to comprise 10% of the initial population of phenotype *T*^−^.

**Table 2:** The coefficients of payoff matrix A (doi:10.1158/1078-0432.ccr-19-0006).

	coefficient values											
	*a*_12_	*a*_13_	*a*_14_	*a*_21_	*a*_23_	*a*_24_	*a*_31_	*a*_32_	*a*_34_	*a*_41_	*a*_42_	*a*_43_
Responder	0.7	0.8	0.7	0.4	0.6	0.5	0.5	0.9	0.8	0.45	0.85	0.95
Non responder	0.7	0.9	0.8	0.4	0.6	0.5	0.5	0.8	0.7	0.45	0.75	0.95

**Table 3:** The initial population densities (doi:10.1016/j.jtbi.2018.09.022).

	Initial population densities			
	*y*_*T*^+^_(0)	$y_{T^{P}}(0)$	*y*_*T*^−^_(0)	*y*_*T*^−−^_(0)
Responder	560.36	747.59	47.10	4.71
Non responder	319.63	707.76	273.97	27.39

The growth of phenotypes is governed by the standard Lotka-Volterra replicator equations [32, 33]:

$$\begin{array}{c} \frac{dy_{i}}{dt}=r_{i}y_{i}(1-\frac{\sum_{j=1}^{4}a_{ij}y_{j}}{K_{i}}) \end{array}$$ (1)

where *y*_*i*_, *r*_*i*_, and *K*_*i*_ represent the population, growth rate, and carrying capacity of phenotype *i*, respectively.

The system of Lotka-Volterra equations (as a generalization of the logistic model for multiple species) is used to model the population dynamics in an ecosystem. These equations are used when several species use limited and shared resources for growth and reproduction.

The growth rate of subpopulations is calculated by determining the doubling time of the study cell lines. The LNCaP cell line (ATCC@CRL-1740) is a *T*^+^ cell line that is dependent on androgens and has a doubling time of 60 hours. The H295R cell line (ATCC@CRL-2128) represents *T*^*P*^ with a doubling time of 48 hours. The PC-3 cell line represents *T*^−^ cells with a doubling time of 25 hours. Using these values, the growth rates of *T*^−^, *T*^*P*^, and *T*^+^ cells were determined to be 0.66542, 0.34657, and 0.27726 per day, respectively. However, these growth rates may not be biologically feasible in a resource-limited tumor environment, so they were scaled to $r_{T^{-}}=6.6542\times 10^{-3}$, $r_{T^{P}}=3.4657\times 10^{-3}$, $r_{T^{+}}=2726\times 10^{-3}$, in our model. The growth rate scaling has no effect on the evolutionarily stable states of the underlying evolutionary game since *r*_*i*_ in 1 is scalable [23].

In the absence of Abiraterone treatment, the values $K_{T^{P}}=K_{T^{-}}=10000$ and *K*_*T*^−−^_ = 6000 have been used for carrying capacity of phenotypes *T*^*P*^, *T*^−^ and *T*^−−^ as well as the other mentioned references. Since the *T*^+^ cheater cells do not need to produce testosterone and get their required testosterone from *T*^*P*^ cells, the carrying capacity of *T*^+^ cells is a multiple of the *T*^*P*^ cell population, i.e., $K_{T^{+}}=\mu \times y_{T^{P}}$, and *μ* is normally considered 1.5. ADT has no effect on the carrying capacity of *T*^−^ and *T*^−−^ phenotypes, but targets the ability of *T*^*P*^ cells to supply testosterone, which in turn reduces the carrying capacity of *T*^*P*^ cells directly and *T*^+^ cells indirectly [9]. Here, consistent with the other mentioned references, during the Abiraterone treatment, for a consumed dose of λ ∈ [0, 1], the values $K_{T^{P}}=10000-9900\lambda$ and *μ* = 1.5 − λ have been used in the Lotka-Volterra equations.

For instance, with a unit dose of Abiraterone, the carrying capacity of *T*^*P*^ and *T*^+^ cells is 100 and $0.5y_{T^{P}}$, respectively. Docetaxel decreases the growth rate of cancer cells (excluding the doubly resistant *T*^−−^ phenotype) and its effect on the Lotka-Volterra equations is shown below:

$$\begin{array}{c} \frac{dy_{i}}{dt}=r_{i}y_{i}(1-\frac{\sum_{j=1}^{4}a_{ij}y_{j}}{K_{i}})\left(1-c_{i}\right) \end{array}$$ (2)

The Lotka-Volterra system, in the context of Abiraterone-Docetaxel treatment, is expressed by the relation presented in 2. The carrying capacity *K*_*i*_ is altered by Abiraterone treatment, and the influence of Docetaxel is reflected in the factor (1−*c*_*i*_) [34–36]. Docetaxel only affects cell growth, i.e. if the population of the i-th phenotype is decreasing, Docetaxel will have no effect on it and *c*_*i*_ = 0. Values greater than 1 for *c*_*i*_ represent the effect of Docetaxel in Eq 2. As noted in mentioned references, we set *c*_1_ = *c*_2_ = *c*_3_ = 1.5 during Docetaxel treatment and *c*_4_ = 0.

Using the replicator equations, we then search for a combination of Abiraterone and Docetaxel treatments that reduces the population of cancer cells and drug-resistant cells to an acceptable level. We define the acceptable level as being below 5% of the carrying capacity for each phenotype.

## Results

In reference [24], the treatment period of 1200 days for Abiraterone-Docetaxel in the best responder group was considered. However, in our study, based on the reference [23], we adopt a longer treatment period of 3000 days for simulating the effect of Abiraterone-Docetaxel treatment on both responder and non-responder groups.

Finding an optimal solution with the minimum number of cancer cells or resistant cells requires a large number of cases to be considered, which is impractical. Simulations were first conducted to identify which tumor subpopulation was under strong selection for various mono and combination therapies. The results showed that the short-term Docetaxel treatment did not significantly reduce cancer cells, and its long-term use in the early stages of treatment caused the rapid growth of resistant cells, making it impossible to reduce them to an acceptable range by the end of treatment. Thus, Docetaxel treatment should be delayed to the last third of the treatment period. The reduction of cancer cells was slow with Docetaxel treatment, so it should start early in the final third of the treatment period to effectively reduce the resistant cancer cell population by the end.

On the other hand, early Abiraterone treatment led to overgrowth of *T*^−^ cells, which could not be reduced without a very long-term administration of Docetaxel. Therefore, Abiraterone treatment should also be postponed to the final third of treatment period and used in conjunction with Docetaxel to control *T*^−^ cells. Additionally, due to Abiraterone’s strong and immediate effect on *T*^+^ and TP cells, a short-term administration of Abiraterone could be used to prevent the growth of resistant cells.

**Fig 1:**
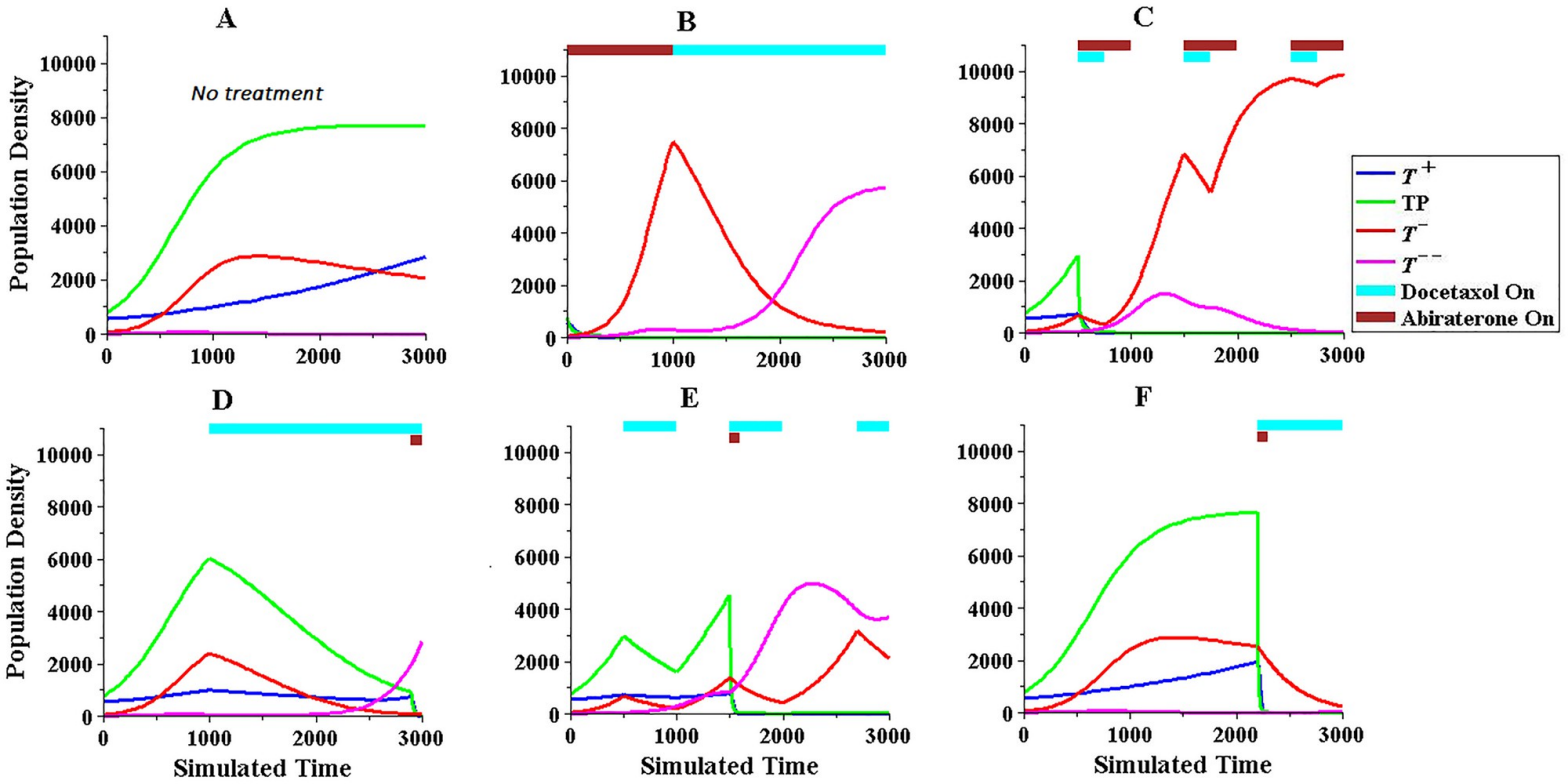
Comparison of some treatment methods on cell population dynamics in responder group with best result shown in 1F.

**Fig 2:**
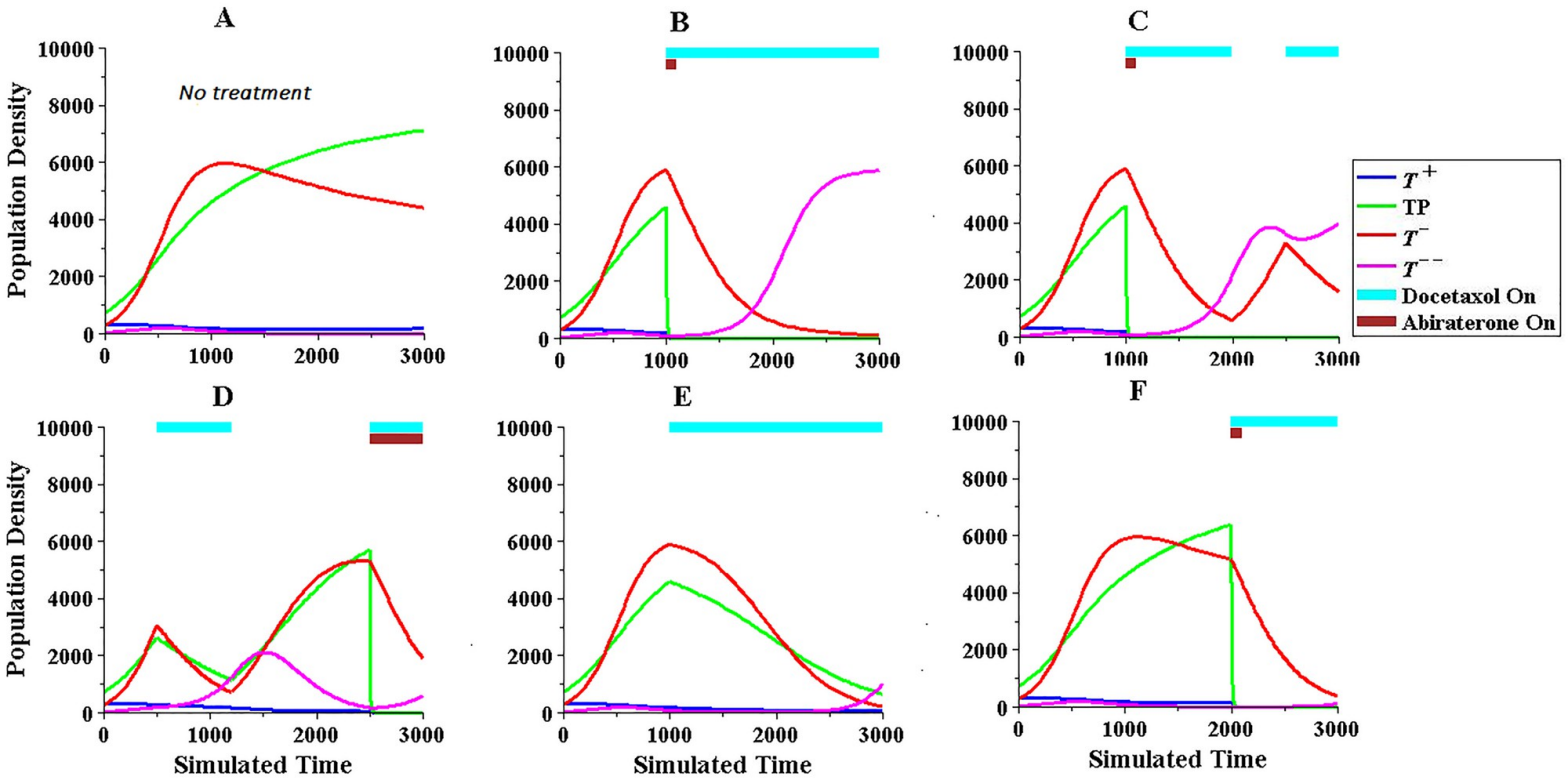
Comparison of some treatment methods on cell population dynamics in non-responder group with best result shown in 2F.

Considering these observations, simulations were performed to find the best combination therapy with acceptable results for both responder and non-responder groups. Some results are presented in Figs 1A to 1F and 2A to 2F, where the treatment methods in Figs 1F and 2F have acceptable results and follow the on-off bang-bang style. The other treatments, in bangbang style, did not yield better results than those in Figs 1F and 2F. The examples in Figs 1 and 2 also suggest that the use of Abiraterone in the responder and non-responder groups should be limited and used in the final third of the treatment to prevent the growth of resistant cells and the use of Docetaxel therapy should also be delayed as much as possible to prevent the growth of phenotype *T*^−−^.

As seen in the figures, the treatments with acceptable results show that the amount of Abiraterone used is low and equal in both responder and non-responder groups. However, the amount of Docetaxel used in the non-responder group is 20% greater than that in the responder group.

## Discussion

Based on the frequency of resistant *T*^−^ phenotypes in ESS, patients with mCRPC are divided into three groups: best responders, responders, and non-responders, both theoretically and practically [9, 19]. Abiraterone treatment with an appropriate schedule is an effective treatment for mCRPC in the best responder group, but in other groups, it causes a cross-sectional decrease in the cancer cell population, leading to rapid growth of drug-resistant cells, which become the dominant subpopulation [19, 23].

Therefore, besides Abiraterone, another treatment must be used for the responder and non-responder groups. In this paper, a combination therapy of Docetaxel and Abiraterone has been considered for these groups. Using the PSA biomarker (which reflects the number of cancer cells) in adaptive therapy is only suitable in the best responder group, where resistant cancer cells are suppressed along with other phenotypes. In other groups, the PSA biomarker overlooks resistant cell growth and results in treatment failure. To provide a combination therapy with acceptable results in the responder and non-responder groups, the extent of changes in triple phenotypes and the doubly resistant phenotype *T*^−−^ (resistant to both Abiraterone and Docetaxel treatments) should be monitored. Simulations showed that in a suitable combination therapy, Abiraterone and Docetaxel can be used in the final third of the treatment in the responder and non-responder groups. This is consistent with the optimal Abiraterone treatment [23], which has advantages such as allowing enough time for tests and decision-making, as well as improving the patient’s quality of life during the first two-thirds of treatment.

Although the combination therapies in this paper reduce the cancer cell population to an acceptable range at the end of treatment (less than 5% of the carrying capacity of each phenotype), the total population of cancer cells during treatment does not effectively decrease and should remain a priority for future research.

The model in this paper was established based on a clinical trial [19], however, additional experimental data is needed to confirm its validity. A challenge in its implementation is selecting the appropriate one of the 22 competition matrices for each patient which needs more research. Currently, standard tests like prostate specific antigen (PSA) for diagnosing and tracking prostate cancer progress are inadequate in determining the frequency of different cancer phenotypes, including drug-resistant ones. To enhance treatment for castration-resistant metastatic prostate cancer, more precise methods for identifying cancer phenotypes and DNA must be established [3, 12, 24, 27, 33]. The rise of new drug-resistant phenotypes can significantly affect the competition’s dynamics and stability [37], therefore further research is necessary to determine if new mutations can emerge and spread within the tumor population [20].

## Supporting information

S1 Table Final population density values and drug administration scheduling related to Fig 1. (PDF)

S2 Table Final population density values and drug administration scheduling related to Fig 2. (PDF)

## References

[1] Wild CP, Stewart BW, Wild C. World cancer report 2014. World Health Organization Geneva, Switzerland; 2014.

[2] Pezaro C, Woo HH, Davis ID. Prostate cancer: measuring PSA. Internal medicine journal. 2014;44(5):433–440. doi: 10.1111/imj.12407 24816306

[3] Chong JT, Oh WK, Liaw BC. Profile of apalutamide in the treatment of metastatic castration-resistant prostate cancer: evidence to date. OncoTargets and therapy. 2018;11:21–41. doi: 10.2147/OTT.S147168 29695920 PMC5905496

[4] Mohler JL, Gregory CW, Ford OH, Kim D, Weaver CM, Petrusz P, et al. The androgen axis in recurrent prostate cancer. Clinical cancer research. 2004;10(2):440–448. doi: 10.1158/1078-0432.CCR-1146-03 14760063

[5] Alex AB, Pal SK, Agarwal N. CYP17 inhibitors in prostate cancer: latest evidence and clinical potential. Therapeutic advances in medical oncology. 2016;8(4):267–275. doi: 10.1177/1758834016642370 27482286 PMC4952018

[6] Sivonova MK, Jurecekova J, Tatarkova Z, Kaplan P, Lichardusova L, Hatok J. The role of CYP17A1 in prostate cancer development: structure, function, mechanism of action, genetic variations and its inhibition. Gen Physiol Biophys. 2017;36(5):487–499. doi: 10.4149/gpb_2017024 29372682

[7] Gomella LG, Singh J, Lallas C, Trabulsi EJ. Hormone therapy in the management of prostate cancer: evidence-based approaches. Therapeutic advances in urology. 2010;2(4):171–181. doi: 10.1177/1756287210375270 21789093 PMC3126080

[8] Sweeney CJ, Chen YH, Carducci M, Liu G, Jarrard DF, Eisenberger M, et al. Chemohormonal therapy in metastatic hormone-sensitive prostate cancer. New England Journal of Medicine. 2015;373(8):737–746. doi: 10.1056/NEJMoa1503747 26244877 PMC4562797

[9] You L, Brown JS, Thuijsman F, Cunningham JJ, Gatenby RA, Zhang J, et al. Spatial vs. non-spatial eco-evolutionary dynamics in a tumor growth model. Journal of theoretical biology. 2017;435:78–97. doi: 10.1016/j.jtbi.2017.08.022 28870617

[10] Montgomery RB, Mostaghel EA, Vessella R, Hess DL, Kalhorn TF, Higano CS, et al. Maintenance of intratumoral androgens in metastatic prostate cancer: a mechanism for castration-resistant tumor growth. Cancer research. 2008;68(11):4447–4454. doi: 10.1158/0008-5472.CAN-08-0249 18519708 PMC2536685

[11] Benzekry S, Hahnfeldt P. Maximum tolerated dose versus metronomic scheduling in the treatment of metastatic cancers. Journal of theoretical biology. 2013;335:235–244. doi: 10.1016/j.jtbi.2013.06.036 23850479

[12] Enriquez-Navas PM, Wojtkowiak JW, Gatenby RA. Application of evolutionary principles to cancer therapy. Cancer research. 2015;75(22):4675–4680. doi: 10.1158/0008-5472.CAN-15-1337 26527288 PMC4693617

[13] Gatenby RA, Silva AS, Gillies RJ, Frieden BR. Adaptive therapy. Cancer research. 2009;69(11):4894–4903. doi: 10.1158/0008-5472.CAN-08-3658 19487300 PMC3728826

[14] Cunningham J, Thuijsman F, Peeters R, Viossat Y, Brown J, Gatenby R, et al. Optimal control to reach eco-evolutionary stability in metastatic castrate-resistant prostate cancer. Plos one. 2020;15(12):e0243386. doi: 10.1371/journal.pone.0243386 33290430 PMC7723267

[15] Staňková K, Brown JS, Dalton WS, Gatenby RA. Optimizing cancer treatment using game theory: A review. JAMA oncology. 2019;5(1):96–103. doi: 10.1001/jamaoncol.2018.3395 30098166 PMC6947530

[16] Gluzman M, Scott JG, Vladimirsky A. Optimizing adaptive cancer therapy: dynamic programming and evolutionary game theory. Proceedings of the Royal Society B. 2020;287(1925):20192454. doi: 10.1098/rspb.2019.2454 32315588 PMC7211445

[17] West J, Hasnain Z, Macklin P, Newton PK. An evolutionary model of tumor cell kinetics and the emergence of molecular heterogeneity driving gompertzian growth. SIAM Review. 2016;58(4):716–736. doi: 10.1137/15M1044825 29937592 PMC6013275

[18] Gatenby RA, Brown JS. Integrating evolutionary dynamics into cancer therapy. Nature Reviews Clinical Oncology. 2020;17(11):675–686. doi: 10.1038/s41571-020-0411-1 32699310

[19] Zhang J, Cunningham JJ, Brown JS, Gatenby RA. Integrating evolutionary dynamics into treatment of metastatic castrate-resistant prostate cancer. Nature communications. 2017;8(1):1–9. doi: 10.1038/s41467-017-01968-5 29180633 PMC5703947

[20] West JB, You L, Zhang J, Brown JS, Gatenby RA, Newton PK, et al. Towards multidrug adaptive therapy. Cancer research. 2020;80(7):1578–1589. doi: 10.1158/0008-5472.CAN-19-2669 31948939 PMC7307613

[21] West JB, You L, Ma Y, Newton PK. Capitalizing on competition: An evolutionary model of competitive release in metastatic castration resistant prostate cancer treatment. Journal of Theoretical Biology. 2018;455:249–260. doi: 10.1016/j.jtbi.2018.07.028 30048718 PMC7519622

[22] Zhang J, Gallaher J, Cunningham JJ, Choi JW, Ionescu F, Chatwal MS, et al. A Phase 1b Adaptive Androgen Deprivation Therapy Trial in Metastatic Castration Sensitive Prostate Cancer. Cancers. 2022;14(21):5225. doi: 10.3390/cancers14215225 36358643 PMC9656891

[23] Cunningham JJ, Brown JS, Gatenby RA, Staňková K. Optimal control to develop therapeutic strategies for metastatic castrate resistant prostate cancer. Journal of theoretical biology. 2018;459:67–78. doi: 10.1016/j.jtbi.2018.09.022 30243754

[24] West JB, Dinh MN, Brown JS, Zhang J, Anderson AR, Gatenby RA. Multidrug cancer therapy in metastatic castrate-resistant prostate cancer: an evolution-based strategy. Clinical Cancer Research. 2019;25(14):4413–4421. doi: 10.1158/1078-0432.CCR-19-0006 30992299 PMC6665681

[25] Wölfl B, Te Rietmole H, Salvioli M, Kaznatcheev A, Thuijsman F, Brown JS, et al. The contribution of evolutionary game theory to understanding and treating cancer. Dynamic Games and Applications. 2022;12(2):313–342. doi: 10.1007/s13235-021-00397-w 35601872 PMC9117378

[26] Archetti M, Pienta KJ. Cooperation among cancer cells: applying game theory to cancer. Nature Reviews Cancer. 2019;19(2):110–117. doi: 10.1038/s41568-018-0083-7 30470829 PMC8557269

[27] Deris A and Sohrabi-Haghighat M. Analysis of cancerous tumor growth by the competitive model based on the evolutionary game theory. International Journal of Nonlinear Analysis and Applications. 2022.

[28] Ploussard G, Terry S, Maillé P, Allory Y, Sirab N, Kheuang L, et al. Class III *β*-tubulin expression predicts prostate tumor aggressiveness and patient response to docetaxel-based chemotherapy. Cancer research. 2010;70(22):9253–9264. doi: 10.1158/0008-5472.CAN-10-1447 21045157 PMC3290526

[29] Smith JM. On Evolution. Edinburgh University Press; 1972. ISBN: 0852242298, 9780852242292.

[30] Gatenby RA, Vincent TL. An evolutionary model of carcinogenesis. Cancer research. 2003;63(19):6212–6220. 14559806

[31] Brown JS. Why Darwin would have loved evolutionary game theory. Proceedings of the Royal Society B: Biological Sciences.2016;283(1838):20160847. 10.1098/rspb.2016.0847 PMC5031650 27605503

[32] Bomze IM. Lotka-Volterra equation and replicator dynamics: a two-dimensional classification. Biological cybernetics. 1983;48(3):201–211. doi: 10.1007/BF00318088

[33] Grossebrummel H, Peter T, Mandelkow R, Weiss M, Muzzio D, Zimmermann U, et al. Cytochrome P450 17A1 inhibitor Abiraterone attenuates cellular growth of prostate cancer cells independently from androgen receptor signaling by modulation of oncogenic and apoptotic pathways. International journal of oncology. 2016;48(2):793–800. doi: 10.3892/ijo.2015.3274 26648519

[34] Simon R, Norton L. The Norton–Simon hypothesis: designing more effective and less toxic chemotherapeutic regimens. Nature Clinical Practice Oncology. 2006;3(8):406–407. doi: 10.1038/ncponc0560 16894366

[35] Simon R, Norton L. The norton-simon hypothesis revisited. Cancer Treat Rep. 1986;70(1):163–169. 3510732

[36] Hofbauer J, Sigmund K, et al. Evolutionary games and population dynamics. Cambridge university press; 1998. ISBN-13:978-0521625708.

[37] Strauss SY. Ecological and evolutionary responses in complex communities: implications for invasions and eco-evolutionary feedbacks. chemotherapeutic regimens. Oikos. 2014; 123(3):257–266. doi: 10.1111/j.1600-0706.2013.01093.x

