# Supplementary material for: Abiraterone-Docetaxel scheduling for metastatic castration-resistant prostate cancer based on evolutionary dynamics
Source: PLoS One. 2023 Mar 9;18(3):e0282646. doi: 10.1371/journal.pone.0282646 (PMC9997888; doi:10.1371/journal.pone.0282646)
Supplement: S1 Table — (PDF) [file pone.0282646.s001.pdf]

S1 Table. Final population density values and drug administration scheduling related to Fig. 1.

Subfigures	1A	1B	1C	1D	1E	1F
Final population densities:						
y_T^+	2815	0	0	742	0	0
y_{TP}	7674	2	0	909	45	3
y_T^-	2055	195	9870	73	3150	235
y_T^{--}	0	5725	21	1939	3935	45
Abiraterone schedule	-	[0,1000]	[500,1000]<br>[1500,2000]<br>[2500,3000]	[2900,3000]	[1500,1600]	[2200,2300]
Docetaxel schedule	-	[1000,3000]	[500,750]<br>[1500,1750]<br>[2500,2750]	[1000,3000]	[500,1000]<br>[1500,2000]<br>[2700,3000]	[2200,3000]
