# Supplementary material for: Abiraterone-Docetaxel scheduling for metastatic castration-resistant prostate cancer based on evolutionary dynamics
Source: PLoS One. 2023 Mar 9;18(3):e0282646. doi: 10.1371/journal.pone.0282646 (PMC9997888; doi:10.1371/journal.pone.0282646)
Supplement: S2 Table — (PDF) [file pone.0282646.s002.pdf]

S2 Table. Final population density values and drug administration scheduling related to Fig. 2

Subfigures	2A	2B	2C	2D	2E	2F
Final population densities:						
y_T^+	178	0	0	0	75	0
y_{TP}	7102	0	0	0	654	0
y_T^-	4401	97	1581	1889	225	373
y_T^{--}	0	5862	3956	597	986	109
Abiraterone schedule	-	[1000,1100]	[1000,1100]	[2500,3000]	-	[2000,2100]
Docetaxel schedule	-	[1000,3000]	[1000,2000] [2500,3000]	[500,1200] [2500,3000]	[1000,3000]	[2000,3000]
